# SERINC as a Restriction Factor to Inhibit Viral Infectivity and the Interaction with HIV

**DOI:** 10.1155/2017/1548905

**Published:** 2017-11-22

**Authors:** Gracia Viviana Gonzalez-Enriquez, Martha Escoto-Delgadillo, Eduardo Vazquez-Valls, Blanca Miriam Torres-Mendoza

**Affiliations:** ^1^Departamento de Disciplinas Filosóficas, Metodológicas e Instrumentales, Centro Universitario de Ciencias de la Salud, Universidad de Guadalajara, Guadalajara, JAL, Mexico; ^2^Laboratorio de Inmunodeficiencias y Retrovirus Humanos, Centro de Investigación Biomédica de Occidente, Instituto Mexicano del Seguro Social, Guadalajara, JAL, Mexico; ^3^Centro Universitario de Ciencias Biológicas y Agropecuarias, Universidad de Guadalajara, Guadalajara, JAL, Mexico

## Abstract

The serine incorporator 5 (SERINC5) is a recently discovered restriction factor that inhibits viral infectivity by preventing fusion. Retroviruses have developed strategies to counteract the action of SERINC5, such as the expression of proteins like negative regulatory factor (Nef), S2, and glycosylated Gag (glycoGag). These accessory proteins downregulate SERINC5 from the plasma membrane for subsequent degradation in the lysosomes. The observed variability in the action of SERINC5 suggests the participation of other elements like the envelope glycoprotein (Env) that modulates susceptibility of the virus towards SERINC5. The exact mechanism by which SERINC5 inhibits viral fusion has not yet been determined, although it has been proposed that it increases the sensitivity of the Env by exposing regions which are recognized by neutralizing antibodies. More studies are needed to understand the role of SERINC5 and to assess its utility as a therapeutic strategy.

## 1. Introduction

The promising and within-reach goal of eradicating acquired immunodeficiency syndrome (AIDS) is not being accomplished. Despite the fact that the number of infected people has stabilized to around 35 million people living with HIV/AIDS, it has not been possible to decrease the numbers of infections since 2010 [1].

The current challenge includes finding a robust vaccine and, on the other hand, controlling infectivity in viral sanctuaries. Through various strategies, there are attempts to avoid viral replication using epigenetics and starting new antiretroviral regimens earlier [2].

Among others, there are proposals that include the endogenous proteins, especially the family of serine-incorporating proteins called SERINC [3]. Within the members of this family, the SERINC5 participates in the defense of the host and has the potential for the development of innovative, antiviral treatments [4].

## 2. Retrovirus and Host Defense

Retroviruses have developed strategies to evade and counteract the host's immune response and achieve successful infection that allows them to spread [5, 6]. Within these capabilities is the blocking of the host proteins that interfere at different stages of the viral cycle [7, 8]. These proteins are called “viral restriction factors” and they seek to counteract the action of viruses by providing a cellular barrier, being the first line of defense against viral infection [9, 10].

The restriction factors are highly conserved and are multitaskers, one of their function is regulated cross-species infections. Consequently, they are less effective against viruses in their natural hosts [10, 11].

In this sense, the human immunodeficiency virus (HIV) is not the exception among retroviruses. The HIV has developed mechanisms to overcome these types of cellular barriers that hinder replication (the restriction factors) through viral proteins that interfere with, or nullify, the activity of the host's defense factors [12, 13]. HIV uses its accessory proteins such as the Vif that counteracts the apolipoprotein B mRNA-editing enzyme 3G (APOBEC3G), Vpr for SLX4 endonuclease complex, Vpu for bone marrow stromal antigen 2 (BST-2) or tetherin, and Vpx for SAM-domain HD-domain containing protein 1 (SAMHD1) [14–19].

Antagonists have been found for all accessory viral proteins except for the negative regulatory factor (Nef). Recently, through Nef-deficient cell cultures, the family of proteins called SERINC was discovered [4, 20]. The SERINC have a high antiviral activity against retroviruses such as lentivirus (HIV, simian immunodeficiency virus (SIV), and equine infectious anemia virus (EIAV)) and gammaretrovirus (murine leukemia virus (MLV)) [4, 21, 22].

## 3. Nef Activity on HIV Virulence

**Figure 1:**
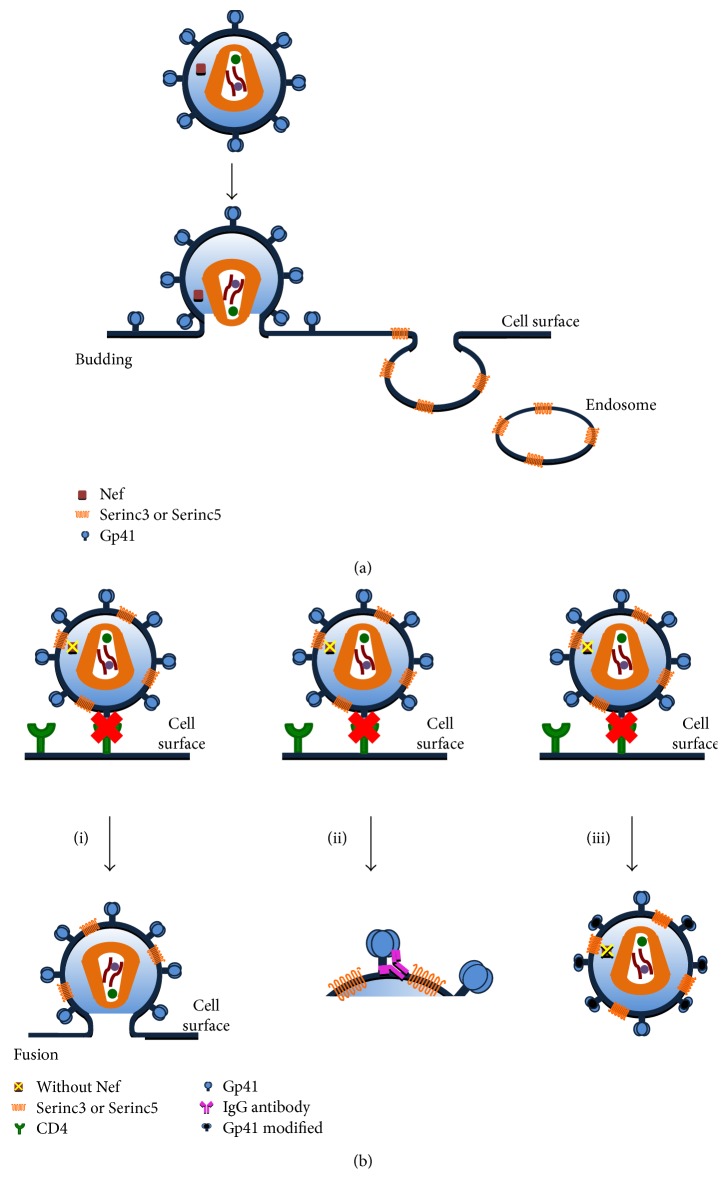
SERINC and Nef interaction in HIV infection. (a) HIV-1 evades the host immune response through manipulation of cell machinery. This process involves the use of vesicular traffic from the plasma membrane to the endosomes and finally be degraded in the lysosome. The HIV uses Nef protein to carry out this activity. In cells infected with viruses expressing the Nef protein, it is observed that SERINC5 was sequestered in the endosomes. (b) HIV-1 with Nef deletion. SERINC5 blocks the activities involved in viral infectivity and does not participate in other Nef-mediated processes. This interaction could have three possible actions and could have as a consequence block or slow the fusion of virus. (i) SERINC5 alters the enlargement of the fusion pore decreasing the ability of the virions to fuse with the target cells; (ii) by slowing the fusion, it would be promoted that gp41 to adopt an open conformation, which would remain exposed for a long time making it susceptible to the neutralizing antibodies; and (iii) SERINC5 could promote structural changes in envelope glycoproteins leading to preventing the entry of the virus prior to small pore formation.

The HIV-1 evades the host's immune response through manipulation of the cell machinery [23]. This process involves the use of vesicular traffic to efficiently direct cell markers such as CD4 and the major histocompatibility complex class 1 (MHC-1) from the plasma membrane to the endosomes, to final degradation in the lysosome [24–27] (Figure 1(a)). To accomplish this activity, the virus uses its Nef accessory protein, which is expressed during the early stages of viral infection [28]. The importance of Nef participation in viral pathogenesis was evidenced by the fact that in SIV, the lack of a functional Nef protein showed a decrease in clinical disease progression and a lower viral load [24, 29].

Nef has a weight of 27–35 kDa and presents myristoylation at the N-terminal end that facilitates association with the cytosolic side of lipids and cell membrane proteins [30]. It has a proline-rich structure that allows it to interact with host tyrosine kinase proteins and AP-2 domain [31–33]. This helps in the recognition of proteins involved in vesicular traffic, such as the family of heterotetrameric adapter protein (AP) complexes, the AP-1, AP-2, phosphofurin acidic cluster sorting protein 1 (PACS1), and the PACS2 [34–39]. The Nef favors the intrinsic infectivity of HIV virions necessary for the full deployment of virus infectivity. The mechanism by which it performs this action is not well defined, yet it is known to cause downregulation of CD4 lymphocytes [31]. The goal is to prevent reinfection and prevent antibody-mediated cytotoxicity; and, it also reduces the MHC-1 to protect infected cells from death by cytotoxic T lymphocytes [40, 41]. Furthermore, it induces the release of virions and contributes to maintaining high viral load in patients [42]. Some patients with deletion of the Nef gene also have downregulation levels of CD4 T lymphocytes, which suggest that it is not the only protein involved in infectivity, although its role is of great importance. In a cohort of 8 individuals infected by transfusion from the same donor who had a deletion of the *nef* gene, they progressed very slowly or did not progress at all, which makes the Nef protein a pathogenic factor [43, 44]. Nef and Vpu contribute to HIV-infected cells evading recognition by the immune system and its consequential cell lysis of cytotoxic T lymphocytes and natural killer cells [45, 46].

## 4. SERINC Family

The SERINC proteins are part of a family of transmembrane proteins present in all eukaryotic cells. These proteins are highly conserved and unique and are not homologous with other eukaryotic proteins, which possibly makes their function in the cell membrane indispensable [3, 47]. The SERINC family is comprised of five members, from SERINC1 to SERINC5, structurally characterized as having 10 to 11 transmembrane domains. SERINC proteins participate in the transport of the serine amino acid through the lipid bilayer and in the biosynthesis of sphingolipids and phosphatidylserine by incorporating serine into membrane lipids [3]. However, the exact physiological function of the SERINC family is still unknown [7, 48].

Of all the members in the SERINC family, only the SERINC3 and SERINC5 have the ability to inhibit viral infection at an early stage of the viral cycle, inhibiting viral fusion and acting as restriction factors [4, 20].

**Figure 2:**
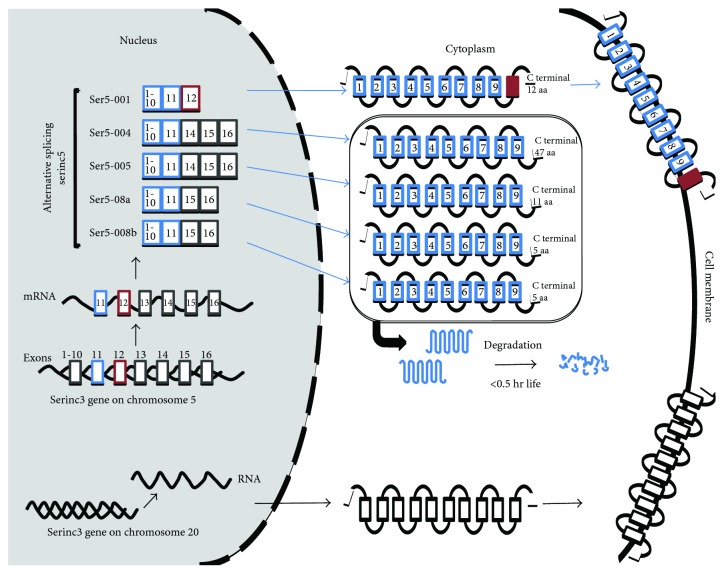
SERINC3 and SERINC5 isoforms. SERINC3 does not have isoforms and is transported directly to the cell membrane. SERINC5 protein has five isoforms generated by alternative splicing. These differ at the terminal carbon end and in the transmembrane domains. The SERINC5-001 isoform has 10 transmembrane domains, only these are in the cell membrane and are involved in the inhibition of HIV infectivity.

The SERINC5 protein has five isoforms generated by alternative splicing (Figure 2). These isoforms have similar topology but differ at the terminal carbon end and in the number of transmembrane domains. Only the SERINC5-001 isoform has 10 transmembrane domains and consequently presents the longest sequence with 461 amino acids, of which 12 are located at their C-terminal end. The remaining isoforms lack the transmembrane domain 10 and have different numbers of amino acids at their C-terminus, located after domain nine: the SERINC5-004, -005, -008a, -008b, and -201 isoforms have 47, 11, 8, 5, and 5 amino acids, respectively. SERINC3 does not have isoforms [49].

The SERINC5-001 isoform is expressed in greater quantities compared to other isoforms. It is the only one that is in the plasma membrane and involved in the inhibition of HIV infectivity. Therefore, the transmembrane domain 10 is key to the SERINC5 activity as a viral restriction factor. The SERINC5-005 and SERINC5-008 isoforms are in the cytoplasm but have a short half-life because they are rapidly degraded. This demonstrates that the 10-domain and the carbon-terminal end are necessary to stabilize and increase the expression of SERINC5 [49, 50].

## 5. Nef Mechanisms to Counter SERINC3 and SERINC5

The antiviral activity of SERINC5 is counteracted by Nef (by leading to a decrease in its incorporation in the virions) because Nef removes it from the plasma membrane and sequesters it in the endosomes for its subsequent degradation [4, 20] (Figure 1(a)). To accomplish this action, Nef requires certain structural characteristics as defined below.

### 5.1. Transport Pathways

Nef induces downregulation of SERINC3 and SERINC5 from the cell membrane by using the cellular transport machinery, mainly using the endolysosomal system and the trans-Golgi network, in a mechanism similar to that used for CD4 downregulation [4, 51–53].

Clathrin-covered vesicles are the main carriers of the endocytic and late secretory pathways, regulating the transport of proteins from the plasma membrane and endosomes to compartments such as other endosomes or lysosomes [54]. Nef sequesters the vesicular traffic of the cells through the modulation of some adapter and accessory proteins involved in the formation of clathrin-coated vesicles [55, 56]. Within these adaptive proteins are the AP complexes and dynamin [57–59]. Nef has conserved sequences such as dileucine motifs (ExxxLL) and carboxy terminal diacid residues (EDAA) that allow it to interact with endocytic machinery, particularly AP1 and AP2 proteins [37, 60, 61]. These motifs are indispensable for the downregulation of MHC-1 and CD4 [51, 57]. Also, Nef requires both regions to antagonize SERINC5 and send it to the endosomes. Therefore, Nef-mediated SERINC5 removal requires the cell to produce AP2 and dynamin [20, 51, 52].

### 5.2. Myristoylation

Myristoylation is a posttranslational change that requires Nef to anchor in the membrane and execute sequestration of the proteins [30, 62, 63]. This modification is carried out in the glycine residue of Nef and when, in experiments, this was substituted by alanine, there was a decrease in the internalization of SERINC5 towards the endosomes for its subsequent degradation [4, 52].

The ability of SERINC3 and SERINC5 to inhibit viral infectivity in cell cultures that had HIV-1 with Nef deletion was demonstrated with genomic and proteomic tools. However, SERINC5 is more potent in its capacity for viral inhibition than SERINC3. SERINC5 reduces wild-type (WT) HIV infectivity in a range between 50% and 90%, whereas SERINC3 only showed a 20% decrease in infectivity [4, 49]. Even when SERINC5 was expressed ectopically, it was observed that the infection was inhibited up to 40 times. SERINC5 inhibition of Nef-defective HIV-1 occurs in a dose-dependent relationship. In contrast, in cells infected with viruses expressing the Nef protein they observed that SERINC5 was sequestered in the endosomes, which prevented their incorporation into viral particles and increased viral infectivity up to 20 to 30 times that observed without Nef-expression cells [4, 20].

When Nef is present, it interacts with SERINC5 to restrict its activity. However, in certain cellular models, the SERINC5 overexpression is able to suppress the capacity of Nef. Thus, it is assumed that Nef activity can be saturated with overexpression of this viral restriction factor [4, 49]. Also, it was found that in few virion-associated SERINC5, the enlargement of the fusion pore is altered, which implies a higher energy spending for pore formation [20] (Figure 1(b)).

The expression of SERINC5 decreases the ability of the virions to fuse with the target cells [4, 20]. SERINC5 only blocks the activities involved in viral infectivity and does not participate in other Nef-mediated processes such as decreased CD4 or MHC-1 [52].

The exact mechanism by which SERINC5 acts to inhibit viral infectivity is still unknown, and there are other elements in addition to Nef that mediate the SERINC5 restriction activity [52]. The type of envelope glycoprotein (Env) that the virus carries is the other determinant able to counteract and resist the SERINC5 action, and this could account why in some cells infected with HIV-1, SERINC5 does not block the fusion [64–66]. Despite *in vitro* experiments not detecting any physical interaction between Env and SERINC5, the presence of virion-associated SERINC5 did not interfere with the incorporation or distribution of Env [65]. So, Nef and Env act by distinct mechanisms to counteract SERINC5 [64].

One possible explanation of the mechanism is that SERINC5 acts to form large oligomers, restricting lipid diffusion and/or inducing hardening of the membranes, leading to decreased mobility of viral particles and interrupting their fusion [65]. The membrane stiffness would slow the folding of the envelope for fusion and promote that the Env would adopt an open conformation, which would remain exposed for a long time and make it susceptible to the neutralizing antibodies [65, 67]. In fact, it was found that the incorporation of SERINC5 increased sensitivity of the antibody 4E10 that targets the membrane-proximal region (MPER) of the gp41, so SERINC5 sensitizes HIV to neutralizing antibodies and inhibitory peptides that recognize conserved gp41 domains [64, 65] (Figure 1(b)).

There are HIV-Env isolates that are resistant to SERINC5. The regions of envelope glycoproteins that contribute to this resistance are located in loops V1, V2, and V3 [66], and this resistance may be impaired when there are inhibitory pressures sensitizing Env to SERINC5 activity [64, 68, 69]. Like when the antiretroviral maraviroc was present, the virions carrying SERINC5 were more sensitive to the maraviroc and neutralizing antibodies [64]. Maraviroc decreases the level of CCR5 receptors on the cell surface that could bind to the Env [70, 71]. This action is potentiated in SERINC5-associated virions by promoting a change in Env conformation that delays the entry of the virus through preventing or slowing the formation of the fusion pore [64]. *In vitro*, SERINC5 causes a conformational change in Env and exposes conserved regions that were identified by the use of neutralizing antibodies [64]. Thus, *in vivo*, subjects infected with certain HIV variants might be more susceptible to maraviroc, for the action of SERINC5, than other subjects with variants resistant to this restriction factor. The greatest susceptibility could be the combination of the conformational changes that generate SERINC5 on Env that delay viral fusion and the recognition of regions conserved through neutralizing antibodies circulating in the subject.

The hypothesis raised by Rosa et al. [4], on the antiviral activity of SERINC5 and its participation in the lipid composition of host cell membranes and HIV-1, was not confirmed by Trautz et al. [4, 48] who found no alterations in the lipid composition of membranes between SERINC5-associated virions with Nef-defective and WT [48]. Although the HIV-1 particles have a higher concentration of saturated lipid species than the cell membranes, the absence of Nef and the presence of SERINC5 did not change this condition [48, 72]. However, a subpopulation of Nef-associated to lipids rafts can alter the lipid composition of this microdomain of the cell host and facilitate signal transduction activities of Nef [73, 74]. In MT-4 T lymphocytes, Nef enhanced sphingomyelin uptake and exclusion of polyunsaturated phosphatidylcholine from the virions, thereby increasing the lipid raft character [73]. In contrast, HIV-1 particles produced from 293 T cells did not show Nef-mediated sphingomyelin enrichment [48]. The diversity reported in the action of Nef may be a consequence of the different cellular models used, similar to the observed variability of SERINC5 to counteract the infectivity of HIV-1 [4]. Another factor is the methodology used, for example, transfecting proviral DNA allowed the Nef-mediated recruitment of Gag into microdomains of 293 T cells; on the contrary, in MT-4 and Jurkat T lymphocytes cells that were not transfected, Gag was not found in lipid rafts [75, 76]. *In vitro* studies do not always correlate with the physiological phenomenon and can yield controversial results [77], so the action of Nef and SERINC5 on the lipid composition should continue to be investigated in different cell models.

## 6. Other Proteins Equivalent to Nef

SERINC5 is fundamental to the restriction of virus infectivity, so those retroviruses that lack the Nef protein count on proteins with a similar activity to block SERINC3 and SERINC5, as is the case with glycosylated Gag (glycoGag) of the gammaretrovirus, MLV, and S2 of the lentivirus EIAV. For this reason, it is considered a potent viral restriction factor [21, 22, 67].

Nef, glycoGag, and S2 do not present structural homology between them, but they have characteristic patterns that allow for inhibiting SERINC3 and SERINC5, since they all have regions of myristoylation and have conserved the domain of dileucine to obtain the union with AP2 [21, 22, 78].

### 6.1. GlycoGag

The glycoGag protein is identical to the Gag protein except for the presence of 88 additional residues at the amino-terminal end. This amino acid sequence acts as a signal to be transported through vesicular traffic to the cell surface to the N-terminal end towards the cytosolic face, and the C-terminal is cut by a protease to be free in the extracellular space [78, 79]. The function of glycoGag has not been completely clarified but it increases the infectivity of MLV particles [80]. The MLV envelope glycoproteins are highly polymorphic and glycoGag has been shown to have a greater contribution to infectivity when certain types of Env that are more sensitive to their action are present [21, 66]. SERINC5 reduces the infectivity of MLV in the absence of glycoGag, and, as with HIV-1, the inhibition is dose-dependent: the greater the amount of SERINC5, the greater the inhibition of MLV [4, 21].

### 6.2. S2

The EIAV expresses the auxiliary protein S2 that has a molecular weight of 7 kDa and is not homologous with other proteins. Its function remained unknown until it was discovered that S2 antagonizes SERINC3 and SERINC5 [22, 81, 82].

The HIV-1 with S2 expression was six times more infectious than the Nef-defective HIV-1. The S2 activity complements but does not add to restoration of the Nef and glycoGag infectivity function. Furthermore, like Nef and glycoGag, S2 has a similar dependence on cell type and envelope glycoprotein to exert its action [21, 22]. The S2 relocalizes SERINC to the endosomes and significantly reduces its incorporation into virions; thus, the S2 executes HIV-1 infectivity by counteracting SERINC3 and SERINC5. Despite the lack of homology that S2 has with others, the Nef, glycoGag, and S2 share two similar sequences, one of which is the site of myristoylation that is located in the glycine of position seven, and the other is the dileucine motif, and both, as mentioned above, counteract the activity of SERINC5 [22, 81]. This points out that the power of SERINC5 to inhibit infectivity is twice that in cells infected with HIV-1 than in cells infected with EIAV envelope glycoprotein [22]. Therefore, the Env type plays a role in the susceptibility of retroviruses to SERINC and participates in the variability of the activity that occurs with different strains of HIV-1, vesicular stomatitis virus (VSV), and the Ebola virus [4, 83].

## 7. Evolution of Nef and SERINC

The fitness to inhibit SERINC5 arose independently in lentiviruses and gammaretroviruses as each developed its proteins to escape the action of this cellular restriction factor [22, 80].

The activity of Nef to counteract SERINC5 is essential for guaranteeing viral infectivity, which is highly conserved among primate lentiviruses, and correlates with the prevalence of these species. Nef mutations that guaranteed highly effective activity against SERINC5 were selected during the adaptation of chimpanzees; in fact, they appear to have gained greater anti-SERINC5 activity after transmission between species of monkeys to apes and from apes to humans [84]. It is also observed that although Nef is one of the most variable proteins among primate lentiviruses, the dileucine motif is highly conserved among species because it is a region that is required to abolish the activity of SERINC5 [52, 84].

SERINC5 has a different evolutionary history than other restriction factors, as it does not present a high frequency of nonsynonymous substitutions in its coding sequence as it occurs with APOBEC3G and SAMHD1. The other functions that SERINC5 performs within the cell may be those that determined its evolution and avoided the arms race or limited it to the introns [85].

## 8. SERINC5: The Promise in HIV

SERINC5 presents new and promising scenarios for both the generation of treatments for HIV infection and the prognosis of the disease. SERINC5 would be as an adjunctive treatment with current antiretrovirals, primarily maraviroc, based on results found in cell cultures where the expression of SERINC5 made the cells more sensitive to the action of three antiretrovirals [64]. To achieve this, SERINC5 would be synthesized by genetic engineering (a fragment that includes the transmembrane domain 10) and incorporated into nanosomes loaded with maraviroc-like signal for delivery to the plasma membrane of the CD4 T cells and counteract the Nef-mediated infectivity*. In vitro* studies have to develop synthetic cell surface receptors that are inserted into the cell surface for the execution of their biologic functions and have the potential of biological drugs [86]. As proteins anchored by glycosylphosphatidylinositol are incorporated in the plasma membrane, they retain native protein function [87]. The use of synthetic cell surface receptors is a better strategy than the gene transfer for the manipulation of the components of the plasmatic membrane [86, 87]. With these strategies, the immunological mechanisms of the host are potentiated by combining the action of restriction factors such as SERINC5 with neutralizing antibodies directed against the MPER of gp41 that guarantee a more successful treatment for the cure and/or eradication of HIV-1 [64, 65].

Levels of SERINC5 expression in serum should be investigated as a possible diagnostic tool to predict response to treatment when using maraviroc. Finally, the results found by Trautz et al. [48] and the evolutionary history of SERINC5 [48, 85] present new questions to elucidate on the SERINC3 and SERINC5 functions within cells and in identifying the biochemical mechanism through which they inhibit viral fusion and the infectivity of retroviruses.
